# Study of the mechanical characteristics of coal-serial sandstone after high temperature treatment under true triaxial loading

**DOI:** 10.1038/s41598-023-40314-2

**Published:** 2023-08-10

**Authors:** Shuai Wang, Lianguo Wang, Bo Ren, Ke Ding, Chongyang Jiang, Jiaxing Guo

**Affiliations:** State Key Laboratory for Geomechanics and Deep Underground Engineering, China University of Mining and Technology, Xuzhou, 221116 China

**Keywords:** Civil engineering, Mineralogy

## Abstract

In this study, a series of true triaxial loading tests were carried out on coal-measure sandstone after high temperature treatment by using a self-developed true triaxial test system combined with acoustic emission (AE) monitoring, and the mass loss, deformation characteristics and loss failure mode of sandstone before and after heat treatment were systematically studied. It is found that the true triaxial mechanical properties of sandstone after high temperature treatment are closely related to temperature, and the peak strength, maximum principal strain, volume strain, minimum fracture angle and elastic modulus, which all showed bimodal changes, and 800 °C is the threshold temperature of the first four parameters. The transition temperature of the elastic modulus is 400 °C. It is found that the test results of true triaxial high temperature sandstone are in good agreement with the existing true triaxial theory and test results. The failure forms of the samples at different temperatures show inverted “Y” or inverted “N” shapes. Shear failure occurs when the temperature is below 400 °C, and shear-tension failure occurs when the temperature is above 600 °C. At the same time, it is found that the AE signal has four periods, namely the quiet period, growth period, explosion period and decline period. The number of AE events corresponds to the deviatoric stress interval well. Experimental study of the mechanical properties of sandstone under the coupling effect of high temperature and true triaxial stress has guiding significance for the parameter selection and safety evaluation of roof sandstone in underground coal gasification.

## Introduction

Due to engineering factors such as the deep-formation disposal of high-level radioactive nuclear waste^1,2^, underground coal gasification and storage^3–6^, and the development of geothermal resources^7,8^, the management of rock-engineering problems in high temperature environments has become an important area of research. Influenced by the composition, microstructure and macrostructure, the physical and mechanical properties of rocks often change significantly after experiencing high temperatures. Because underground rock-engineering activities are usually carried out under true triaxial stress^9,10^, it is very important to study the strength, damage and failure behavior of rocks under true triaxial stress to accurately predict and evaluate the stability of underground rock engineering.

There are many differences in the mineral composition of rocks due to the physical and chemical processes involved in diagenesis. The thermal expansion rate of different mineral particles is different. Under the action of the thermal load, microcracks will close, expand, and condense in rock samples^11–13^. To date, scholars at home and abroad have carried out extensive research on the physical properties of high-temperature-treated sandstone, and achieved fruitful results. The researchers found changes in the thermal properties of the high-temperature-treated sandstone, including the mineral composition^14,15^, porosity^16,17^, wave velocity^18,19^, and thermal conductivity^20^. These changes affect the physical and mechanical properties of rocks, so it is important to further understand the thermal damage behavior of rocks.

The real-time physical and mechanical properties testing of rocks at a high temperature is limited by the availability of equipment. Therefore, scholars usually treat rocks with a high temperature first, and wait for the temperature to drop to normal temperatures before conducting various tests. Additionally, researchers have carried out laboratory mechanical tests on high-temperature sandstone. Uniaxial compression tests^21–24^, Brazilian splitting tests^25,26^, shear tests^27^ and conventional triaxial tests^28,29^ were carried out to test compression strength, tensile strength, peak strain, elastic modulus, Poisson's ratio, cohesion, the internal friction Angle and other parameters of high-temperature-treated sandstone, and to discuss the correlation between stress and high temperatures. There are different views in the research of temperature thresholds. Some scholars believe that major changes in rock strength usually occur at above 600 °C^30,31^, while others believe that the rock structure will also soften when the temperature is 500–600 °C^32,33^. It was found that these changes were mainly attributed to the transformation of the crystal structure, mineral decomposition, and water loss^34,35^. For sedimentary rocks such as sandstone, the coefficient of thermal expansion mainly depends on its mineral composition; for example, a higher occurrence of damage is likely under high temperatures when the rock contains higher levels of calcite^36^. The physical and mechanical properties of some sandstones after heat treatment are summarized in Table 1.

**Table 1:** Effect of thermal treatment on the physical and mechanical properties of sandstone: insights.

Ref	Site	Max. temp. achieved (°C)	Measurement condition	Main mineral	Tested properties
Ranjith et al.^22^	Gosford, New South Wales, Australia	950	Cooled down	Quartz, Kaolinite, Illite	UCS, Elastic modulus
Zhang et al.^32^	Xuzhou, China	800	Heating process	–	UCS, Elastic modulus
Hajpál^18^	German and Hungarian	900	Cooled down	Quartz, Feldspar, Micas, Clay minerals (Kaolinite, Chlorite, Glauconite)	Density, Porosity, Water adsorption, Ultrasonic sound velocity, Duroskop rebound, UCS and Indirect tensile strength
Sun et al.^20^	Linyi, China	900	Cooled down	Quartz, Feldspar, Dolomite/Ankerite, Hematite/Magnetite	Thermal conductivity, Thermal diffusivity, Heat capacity
Lei et al.^21^	Chongqing,China	900	Cooled down	Quartz, Feldspar, Plagioclase, Clayminerals	Mass, Volume, Density, Poisson’s ratio, Elastic modulus, UCS
Mahanta et al.^37^	Rajasthan, India	900	Cooled down	Quartz, Dickite, Kaolinite, Calcite	UCS, Tensile strength, Elastic modulus, Poisson’s ratio

To be able to characterize the progressive rupture behavior of high-temperature sandstone triaxial tests, researchers have investigated sandstone damage by means of acoustic emission^38,39^, CT radiography^40^, infrared thermography^41^ and nuclear magnetic resonance techniques^42^. However, the existing research on the damage evolution and failure behavior of high temperature sandstone mainly focuses on uniaxial compression^43^, conventional triaxial states, and the Brazilian splitting test. The deformation characteristics, strength characteristics, and acoustic emission monitoring law of high temperature rocks under true triaxial tests (TTT) require further investigation. The origin of the true triaxial tests can be traced back to 1971, since Mogi^9^ developed the world's first true triaxial test machine, which opened the prelude to the true triaxial test and proposed the octahedral strength criterion for rocks, Haimson et al.^44–46^ further discovered the effect of intermediate principal stress on specimens, Al-Ajmi et al.^47,48^ found a better fit for the linear octahedral strength criterion by collating a large amount of true triaxial data, and called their strength criterion the Mogi-Coulomb criterion.

The aim of this study is to reveal the intrinsic mechanism of temperature effects on rock strength. Firstly, the effect of temperature on the physical and mechanical properties of sandstone was analyzed. The mineral composition of coal-serial sandstone was analyzed using X-ray diffraction (XRD) method. Secondly, true triaxial loading tests were carried out on sandstone samples subjected to high temperatures using a self-developed true three-axis electro-hydraulic servo loading test system^49^, and the causes and regularity of changes in the mechanical parameters of sandstone following high-temperature treatment were analyzed. During the tests, an acoustic emission monitoring system was used to capture the damage evolution and failure process of sandstone in real time^50^. The aim of this paper is to provide a scientific basis for predicting and evaluating the stability and safety of underground engineering, a high-temperature sandstone roof for underground coal gasification.

## Rock sample preparation and test methods

### Experiential materials

**Figure 1:**
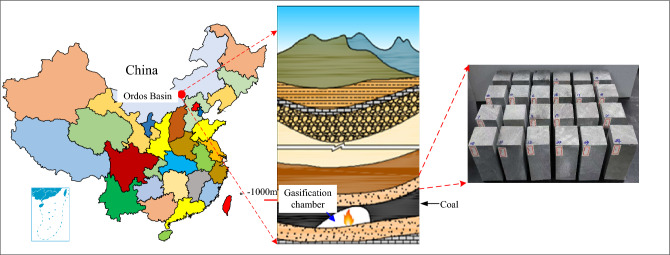
Schematic diagram of the sandstone sampling location.

**Figure 2:**
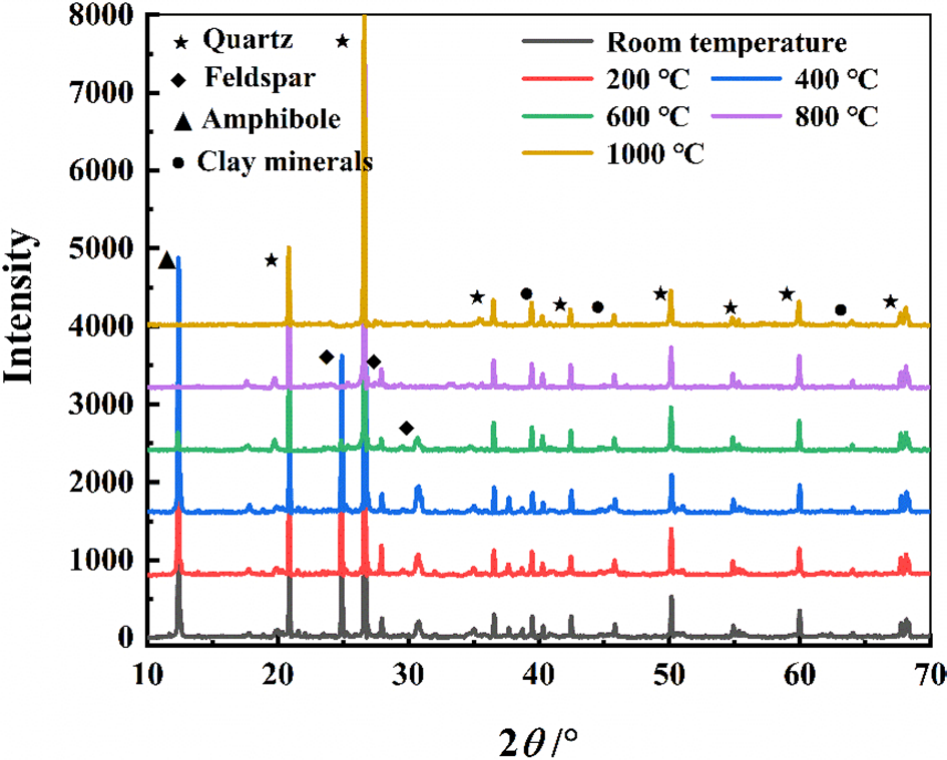
XRD analysis of sandstone.

The experimental rock samples are taken from the roof sandstone of the Taiyuan Formation–Shanxi Formation coal seam in Ordos Basin, and the rock mass is hard and compact^15^. The sampling position of the test rock sample is shown in Fig. 1. The rock formation is at 950–1020 m below ground. Macroscopically, the hue of sandstone at NT is gray, and the outer surface is smooth without obvious holes, joints or macroscopic cracks. The results of X-ray diffraction analysis revealed that the rock samples were feldspar quartz sandstone, containing 44% quartz, 35% feldspar minerals, 10% clay minerals, 7% calcite, and 4% zeolite^51^ (as shown in Fig. 2). According to the requirements of the test platform, the processed rock sample is a rectangular specimen with length, width and height of about 50 mm × 50 mm × 100 mm. The average density of the sample at normal temperature (NT) is 2620 kg/m^3^. According to field studies on underground coal gasification, the use of non-well, reverse ignition results in the formation of a void known as the gasification chamber, where a combustion zone is formed. The temperature of the flame working face can normally reach 800–1200 (°C) during gasification. The thermal energy in the working face can be transferred to surrounding rock through thermal conduction, convection, and radiation, resulting in changes in the temperature field within surrounding rock^52^. Based on different experimental temperatures, the samples were divided into six groups (a total of 24 sandstone specimens, with an additional six specimens). In order to analyze and compare the effects of temperature on the micro-damage of the sandstone, scanning electron microscopy was used to observe the micrographs of the sandstone specimens after exposure to high temperatures.

### Experimental equipment

#### MXQ1700 box type atmosphere furnace

MXQ1700 box-type atmosphere ovens^15^ (produced by Shanghai Weixing Machinery Equipment Co., Ltd., China) use a Japanese SMC digital display pressure gauge, which has high temperature control accuracy, a temperature control accuracy of 1 °C, average temperature in the oven of 5 °C, and the highest heating temperature can reach 1700 °C. At the same time, the equipment has a built-in water cooling system and vacuum system, and the vacuum degree can reach 0.1 MPa. It can meet the high-temperature heating requirements of sandstone on the roof of deep underground coal gasification coal seam.

#### True triaxial electro-hydraulic servo loading test system

**Figure 3:**
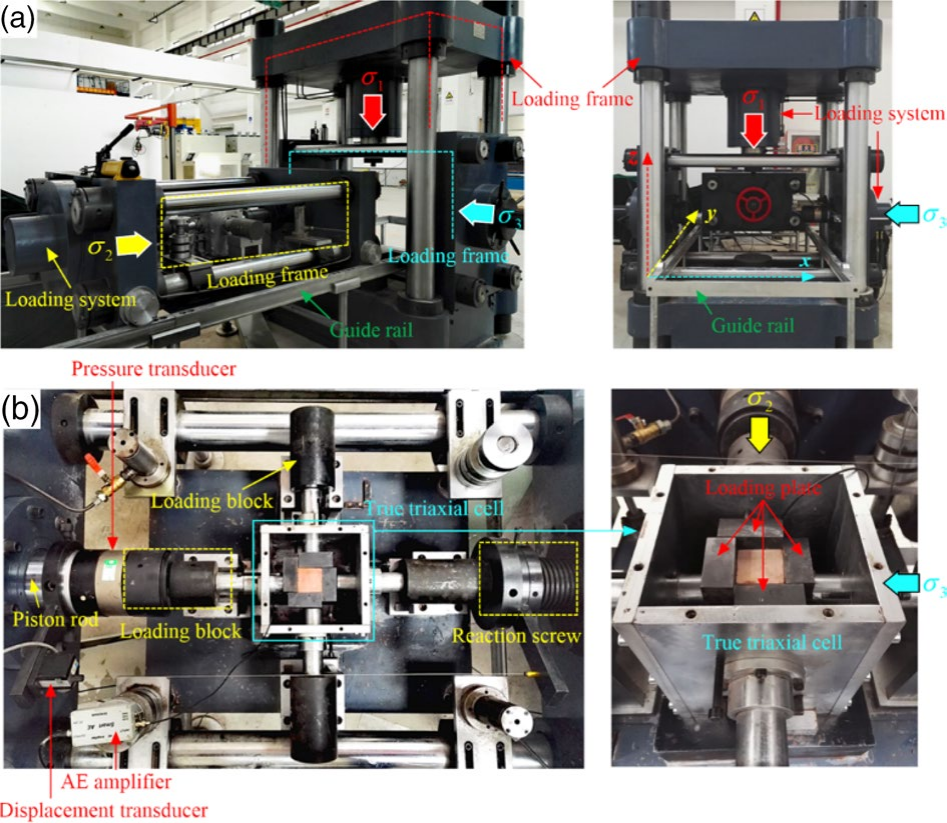
True triaxial compression test system^49^.

This test was conducted on a self-developed rock true triaxial electro-hydraulic servo loading test system, which mainly comprised a triaxial servo control loading system, a true triaxial pressure chamber, and an automatic acquisition system (Fig. 3)^49,53^.

### Experimental process

#### Heating process of sample

**Figure 4:**
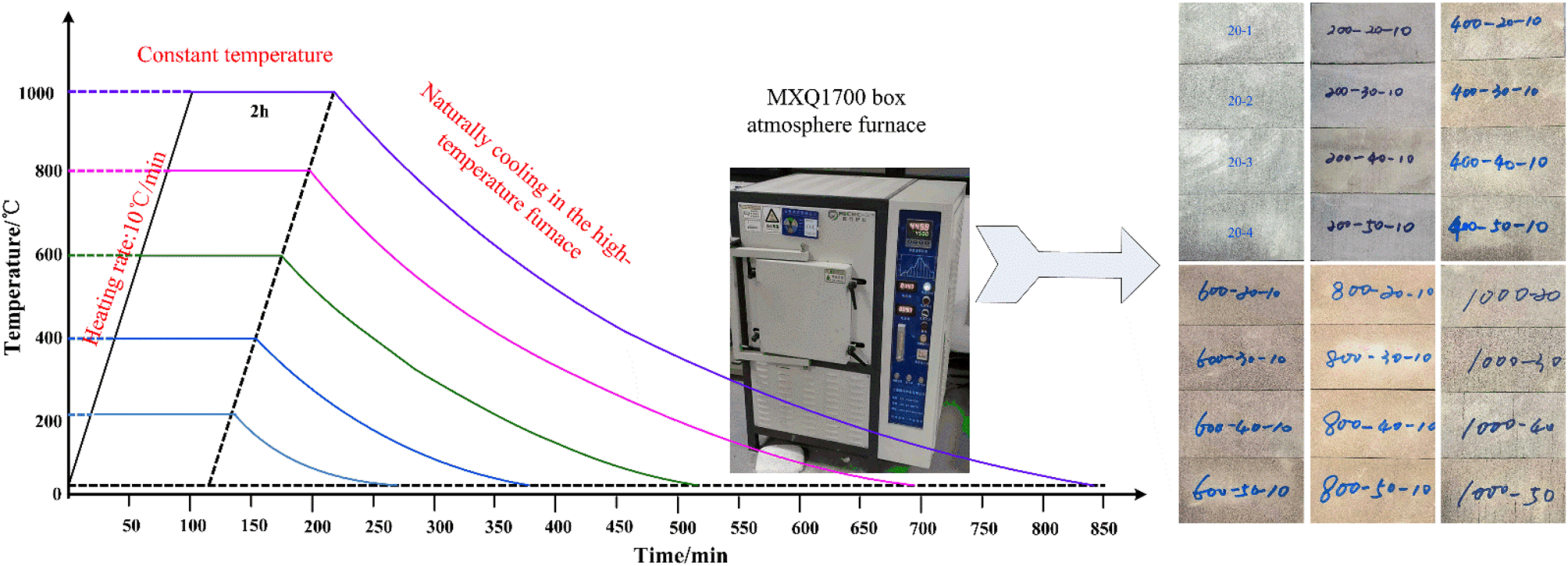
Curves of sandstone samples in the heat treatment^15^.

The heating curve of the sample is shown in Fig. 4. Following high-temperature treatment, the rock samples change from gray to brown–red, and the color gradually deepens with the increase in temperature; however, the sandstone samples at 1000 °C become dark and produce transverse cracks perpendicular to the height direction. The rock sample grouping and basic parameters are shown in Table 2.

**Table 2:** Physical parameters of rock samples.

Sample temperature	Sample no	Length/mm	Width/mm	Height/mm	Weight/g		Mass loss/g
					Before heating	After heating	
NT	SD-20-1	49.8	50.1	100.1	644	644	0
	SD-20-2	49.9	49.9	100.2	647	647	
	SD-20-3	50.2	50.1	99.9	651	651	
200 °C	SD-200-1	50.0	50.1	100.0	645	641	3.67
	SD-200-2	50.1	50.0	100.1	648	644	
	SD-200-3	49.9	49.8	100.0	651	648	
400 °C	SD-400-1	49.8	50.0	100.1	645	642	4.33
	SD-400-2	49.8	49.9	99.9	648	642	
	SD-400-3	49.7	50.1	100.1	654	650	
600 °C	SD-600-1	49.8	50.0	100.2	646	632	13.33
	SD-600-2	49.9	50.1	100.1	648	635	
	SD-600-3	49.8	50.2	99.8	657	644	
800 °C	SD-800-1	49.9	49.9	100.0	646	614	30.33
	SD-800-2	49.8	49.9	100.1	648	620	
	SD-800-3	49.9	49.9	100.2	657	626	
1000 °C	SD-1000-1	50.0	50.1	100.1	647	597	48.67
	SD-1000-2	50.1	50.1	100.1	649	606	
	SD-1000-3	50.1	50.2	100.2	658	605	

#### Loading test scheme

**Figure 5:**
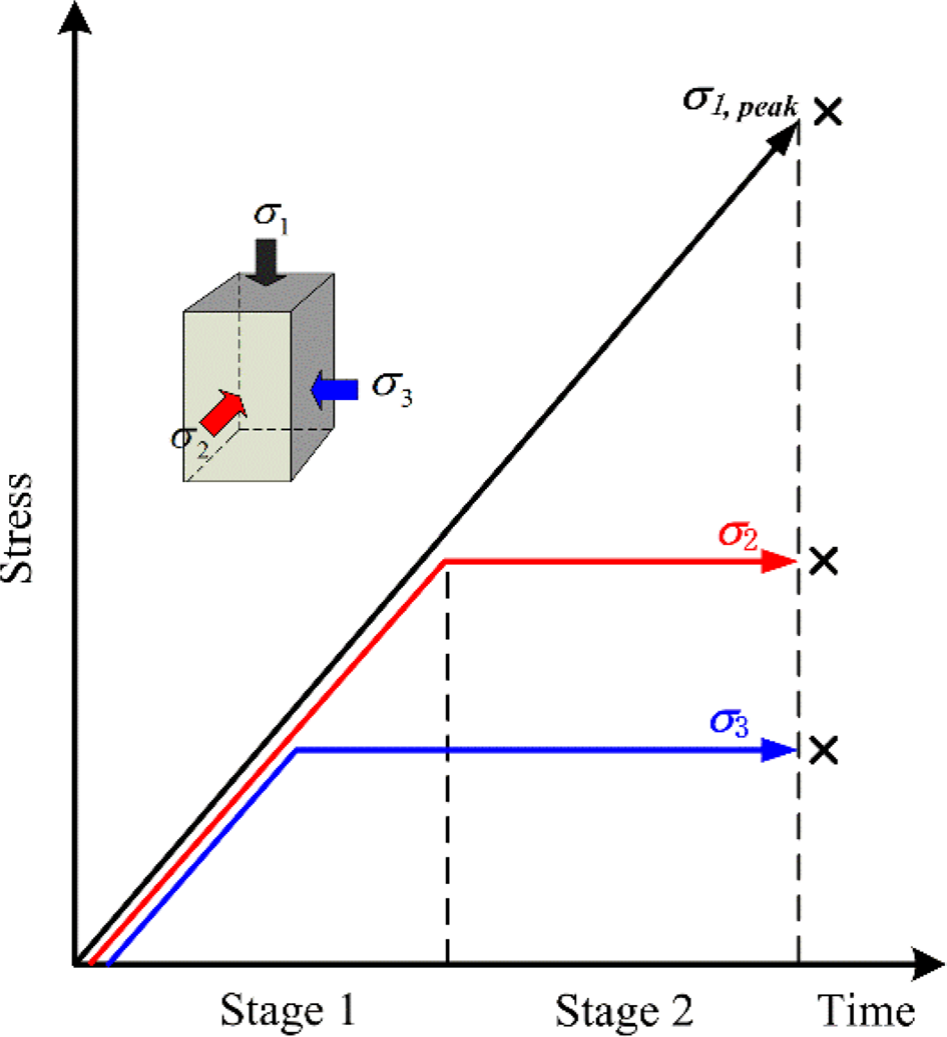
Schematic diagram of loading stress paths.

The deep rock mass is in a three-dimensional unequal compressive stress state. In order to simulate a more realistic stress environment, the loading scheme is shown in Fig. 4. The specific steps are described in the following (Fig. 5)^54^.

1. Apply a certain preload to fully fix the sample, and then apply the confining pressure to the hydrostatic pressure state at a rate of 0.04 MPa/s. At this time, $\sigma_{1} = \sigma_{2} = \sigma_{3} = 10\;{\text{MPa}}$.
2. With $\sigma_{3}$ kept unchanged, continue to increase $\sigma_{1}$ and $\sigma_{2}$ at the same rate to 20 MPa.
3. With $\sigma_{2}$ and $\sigma_{3}$ kept unchanged, increase $\sigma_{1}$ at a rate of 0.004 mm/s according to the displacement loading method until the sample fails. During the test, the AE activities are recorded in real-time.

## Results

### Analysis on deformation characters

**Figure 6:**
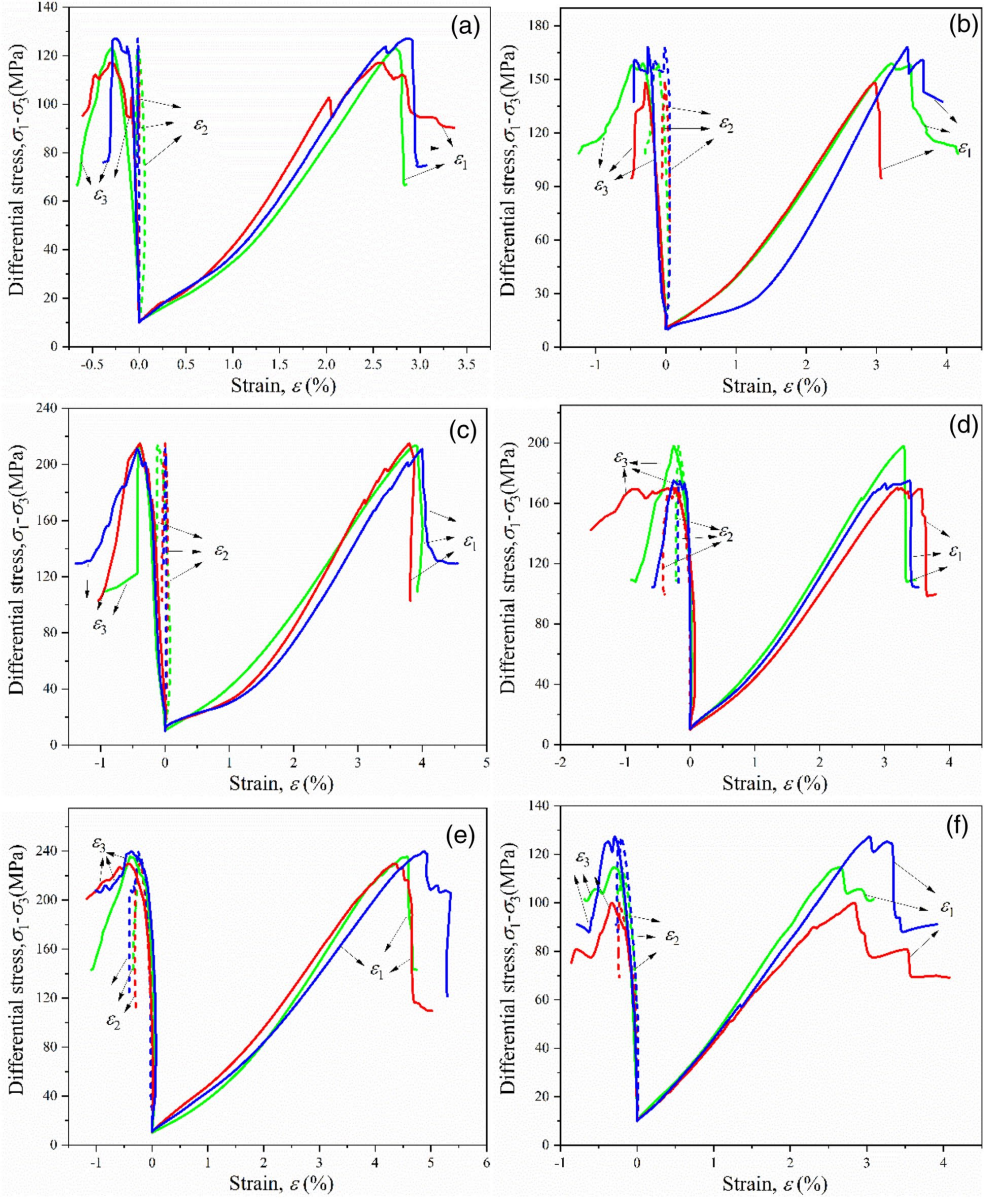
The relationship between deviatoric stress and the three principal strains under true triaxial conditions is shown. Each color represents a single experiment, and figures (**a**)–(**f**) represent the experimental data at six different temperatures.

The stress–strain curve plays a crucial role in understanding the mechanical behavior, deformation characteristics, and strength properties of sandstone specimens. The stress–strain curves of sandstone samples under the true triaxial loading stress paths are illustrated in Fig. 6. In a three-dimensional stress state, the deformation of an object can be described by three principal strains, denoted as *ε*_1_, *ε*_2_, and *ε*_3_, which represent the deformations occurring along the three principal strain directions.

The true triaxial stress–strain curve of the sandstone exhibits four typical stages, namely the upward concave segment, linear segment, downward bending slope segment, and post-peak strain softening stage. Within the temperature range of room temperature to 800 °C, there is a significant stress reduction after the peak, indicating that the sandstone in the underlying formation still exhibits a strong brittle behavior. When the temperature increases to 1000 °C, the sample undergoes less stress reduction after the peak. The sandstone sample shows a clear trend of plastic flow.

The relationship curves between deviatoric stress and the three principal strains in sandstone exhibit similarities under different temperatures. All curves demonstrate non-linear characteristics. Due to the different true triaxial stress states, *σ*_3_ is the smallest; therefore, the strain in that direction (minimum principal strain *ε*_3_) exhibits expansive deformation characteristics. The post-peak stage is entirely characterized by softening, and when the temperature increases to 1000 °C, the maximum principal strain shows a post-peak plastic flow trend.

In conclusion, all instances of the failure of sandstone samples in the range of NT to 800 °C show sudden brittle failure located at the peak stress point. When the temperature reaches 1000 °C, the ductility of the sandstone specimens is enhanced, there is no sudden stress drop, and a plastic flow trend is observed.

**Figure 7:**
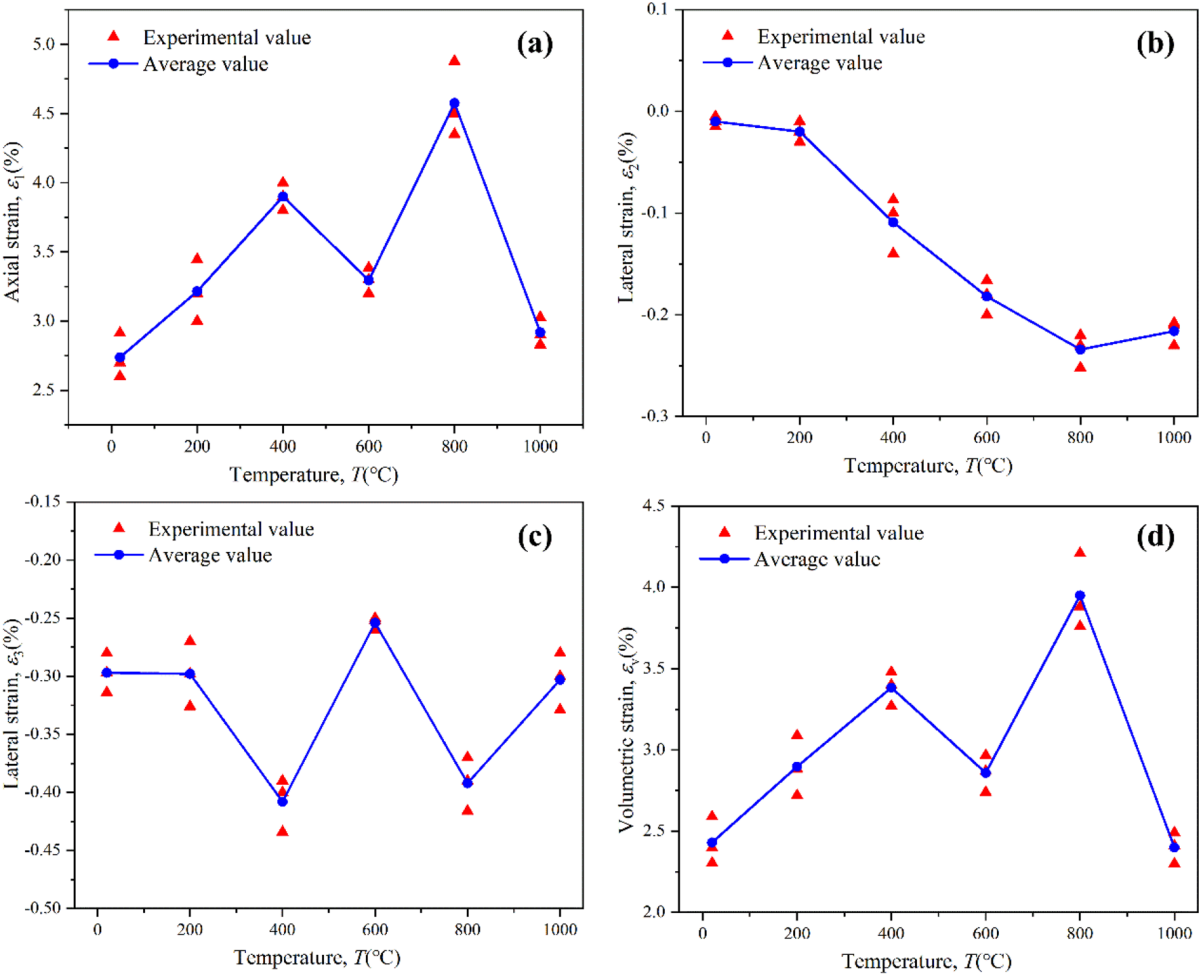
The variation of principal strain with temperature under true triaxial conditions; (**a**) maximum principal strain; (**b**) intermediate principal strain; (**c**) minimum principal strain; and (**d**) volumetric strain.

In order to better analyze the influence of temperature on deformation characteristics, the relationship between principal strain and temperature is obtained, as shown in Fig. 7. A positive value indicates compressive deformation, while a negative value indicates expansion deformation. The peak strain refers to the maximum strain reached by a material under loading or stress. In true triaxial tests, since there are three principal directions, three peak strain values will be obtained for each direction. The peak strain (*ε*_1_ direction) exhibits a "bimodal" pattern with increasing temperature (Fig. 7a). The maximum principal strain is the most significant and plays a decisive role in the three principal stress directions. When the temperature rises from NT to 400 °C, the maximum principal strain increases from 2.74 to 3.90%, representing an increase of 42.3%. From 400 to 600 °C, the maximum principal strain of sandstone decreases from 3.90 to 3.23%, with a decrease of 17.2%. When the temperature ranges from 600 to 800 °C, the maximum principal strain of sandstone increases from 3.23 to 4.58%, with an increase of 41.8%. When the temperature ranges from 800 to 1000 °C, the maximum principal strain of sandstone drops sharply from 4.58 to 2.92%, with a decrease of 36.4%. Compared with the normal temperature, the maximum principal strain at 1000 °C is equivalent, with an increase of only 6.5%. In short, the maximum principal strain of sandstone has two peaks at 400 °C and 800 °C, which are closely related to the stress state.

The intermediate principal strain exhibits a unimodal change with increasing temperature (Fig. 7b). As the peak strain is negative, it indicates that the intermediate principal strain undergoes expansive deformation. The absolute value of the peak strain increases and then decreases with increasing temperature. The threshold temperature is 800 °C. In this experiment, the intermediate principal stress plays a role in constraining the deformation of the specimen in that direction. The absolute value of the peak strain in the ε_2_ direction of the sandstone ranges from 0.1 to 0.25%, indicating relatively small values. The peak strain in the direction of the minimum principal stress (Fig. 7c) exhibits a bimodal change with increasing temperature, similar to the pattern shown in Fig. 7a. However, the numerical values of the peak strain in the direction of the minimum principal stress are negative, indicating that the specimens undergo expansive deformation in that direction during the failure stage. It should be noted that the two turning points of the peak strain occur at 400 °C and 800 °C, and the strain values are relatively close.

Volumetric strain is the sum of the three principal strains in different directions. The formula (Eq. 1) for volumetric strain is as follows:

1 $$ \varepsilon_{{\text{v}}} = \Delta V/V_{0} = \varepsilon_{{1}} \; + \;\varepsilon_{{2}} \; + \;\varepsilon_{{3}} $$

Δ*V* refers to the volume change during compression of the specimen, while *V*_0_ represents the initial volume of the specimen without any applied stress.

Since the direction of the maximum principal stress plays a crucial role and the deformation in the direction of the maximum principal strain is significant, the relationship between volume strain and temperature (Fig. 7d) is controlled by the maximum principal strain, exhibiting a similar pattern as shown in Fig. 7a. The maximum value of the volume strain is 4%, corresponding to 800 °C, indicating that the sandstone can withstand significant volumetric deformation at this temperature. This demonstrates that the deformation characteristics of the coal-bearing sandstone exhibit a strong temperature effect.

**Figure 8:**
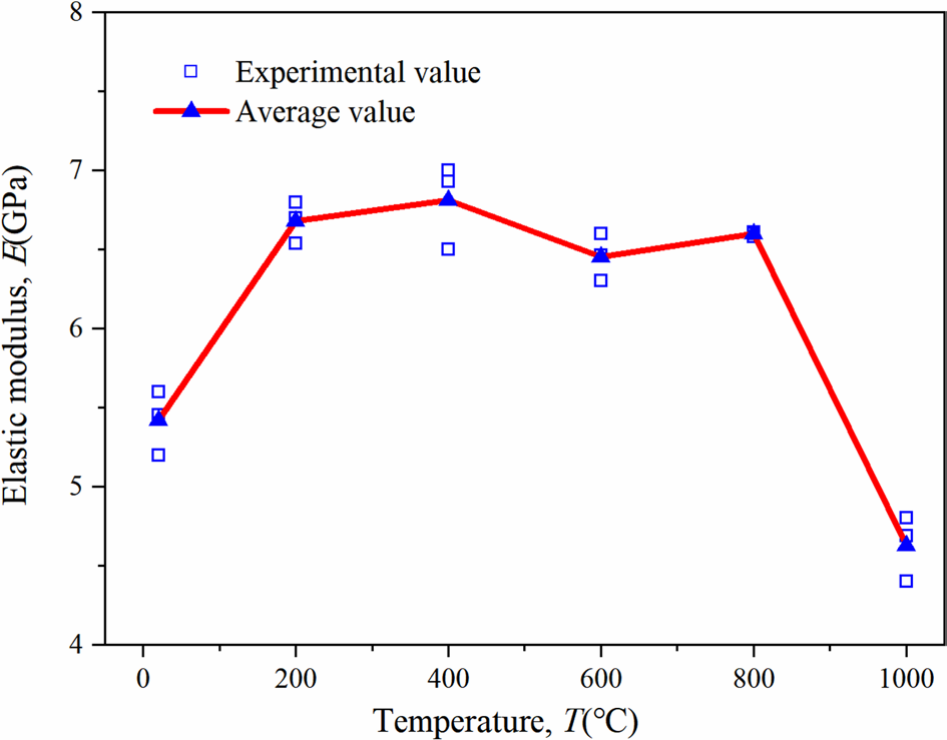
Temperature dependence of modulus of elasticity of sandstone.

According to the International Rock Mechanics Recommendation^55,56^, the slope of the straight line segment in the stress–strain curve is taken as the elastic modulus data, and the relationship between the elastic modulus of sandstone and temperature is shown in Fig. 8. The elastic modulus increases first and then decreases with the increase in temperature, showing a bimodal phenomenon. The elastic modulus of sandstone is the highest at 400 °C. When the temperature rises from NT to 400 °C, the elastic modulus increases from 5.42 to 6.81 GPa, representing an increase of 25.6%. When the temperature ranges from 400 to 600 °C, the elastic modulus decreases from 6.81 to 6.45 by 5.2%. After reaching 600–800 °C, the elastic modulus of sandstone increases slightly. From 800 to 1000 °C, the elastic modulus of sandstone suddenly drops from 6.60 to 4.63 GPa, with a decrease of 29.8%. Compared with normal temperature, the elastic modulus of sandstone decreases by 14.5% at 1000 °C. In short, the elastic modulus of sandstone reaches the maximum at 400 °C, changes only slightly in the range of 200–800 °C, and drops sharply when the temperature exceeds 800 °C.

### Analysis on strength characters

**Figure 9:**
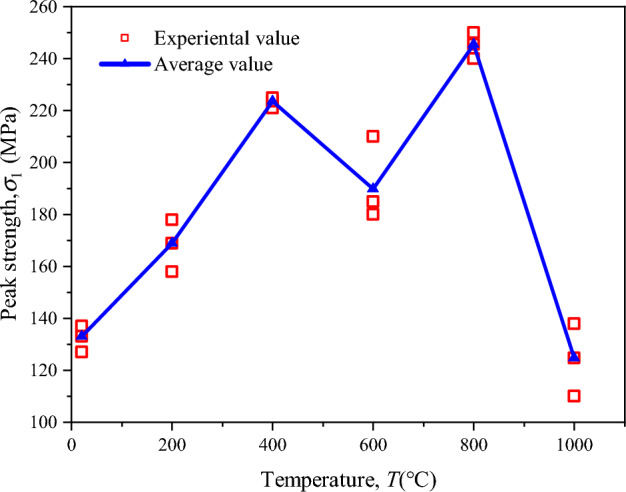
Temperature dependence of peak stress.

The effect of temperature and stress coupling on the peak strength of sandstone under the true triaxial condition is shown in Fig. 9. It can be seen that with the increase in temperature, the average peak strength of sandstone presents a bimodal variation law (Fig. 9). There are two extremes at 400 °C and 800 °C. The strength of sandstone increases continuously from NT to 400 °C, with an increase of 73.5%. After the temperature continues to increase, the strength decreases for the first time, with a decrease of 15.8%. The average peak strength of sandstone reaches the maximum value of 245.45 MPa at 800 °C, and then decreases sharply at 1000 °C, with a decrease of 49.2%.

### AE behavior

**Figure 10:**
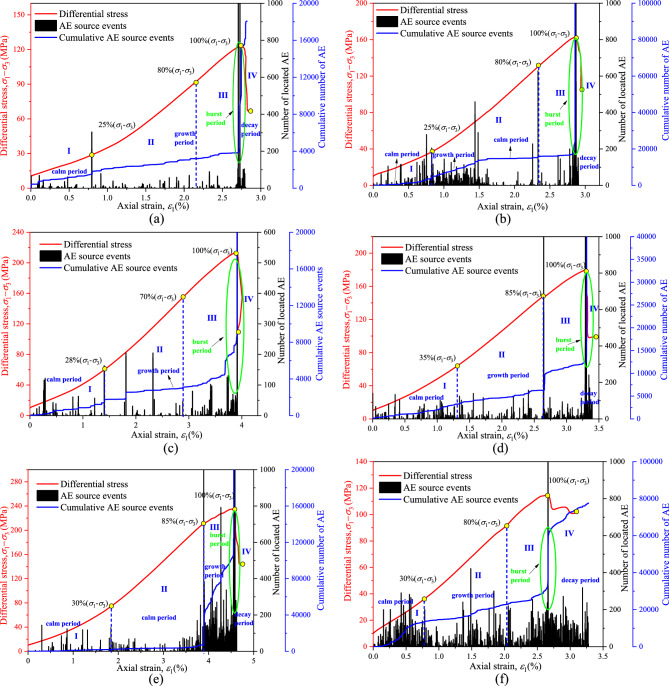
Variation in the differential stress and number of acoustic emission events with axial strain under true triaxial conditions at different temperatures. (**a**) NT, (**b**) 200 °C, (**c**) 400 °C, (**d**) 600 °C, (**e**) 800 °C and (**f**) 1000 °C.

Rock acoustic emission (AE) is the phenomenon of transient elastic waves caused by lattice dislocation or micro-crack propagation in the process of the loading and deformation of rock materials. Previous studies have shown that micro-cracking is the main cause of acoustic emission activity of sandstone^57^. Figure 10 shows the number of AE events and the variation of differential stress with axial strain during the failure of sandstone specimens under TTT conditions (*σ*_3_ = 10 MPa and *σ*_2_ = 20 MPa).

From the above test results, it can be seen that the sample has obvious acoustic emission phenomenon in the test, and the number of acoustic emission is taken as the index of acoustic emission activity in the analysis. According to the stage division of the damage evolution process of sandstone in the true triaxial state, stage II (stable propagation stage of microcracks) begins with the first significant increase in stress in the acoustic emission signal, which is about 25–35% of the peak differential stress; Stage III (unstable microcrack propagation stage) begins with the second sharp increase in the acoustic emission signal stress^58,59^, which is about 70%-90% of the peak differential stress. When the stress reaches 25–35% of the strength limit, the acoustic emission activity becomes clearly strengthened for the first time, which indicates the generation of microscopic cracks in rock. When the stress reaches 70–90% of the strength limit, the acoustic emission counting rate reaches the maximum, which leads to the appearance of fracture surfaces in rock.

Acoustic emission cumulative counts exhibit distinct stages, which can also be divided into the following four periods based on the slope changes of the cumulative curve: calm period, growth period, burst period, and decay period. The initial compaction stage is the quiet period. There are different degrees of AE activities, but there are few AE events. The early compaction stage can be understood as the gradual closure of some original cracks in rock under this low stress level, and AE events will occur during the closure process, but the energy is not high, and it has great randomness and fluctuation. After entering the elastic stage, the acoustic emission activity is still relatively low. However, later in the elastic stage, the activity of AE gradually increases, and occasionally a larger level of AE is produced. With the gradual increase in stress, the activity of AE gradually increases, resulting in some AE events with higher energy, mainly because cracks in some areas inside the specimen begin to slip, referred to as the growth stage. With the increase in the load, cracks in the specimen are gradually produced, and more acoustic emission phenomena are produced. When the peak intensity is reached, most of the acoustic emission numbers reach the maximum value, which is called the burst period. As the specimen continues to compress into the residual strength, the acoustic emission rate decreases gradually, indicating that macroscopic cracks have appeared in the specimen, and this stage is part of the decline stage. However, as can be seen from Figure f, there are many events in the quiet period of the sample after a 1000 °C high temperature, which indicates that the cracks on the rock surface expand further when they begin to compress, and gradually form new cracks. When multiple cracks form and penetrate gradually, a macroscopic fracture surface is finally formed.

### Macroscopic fracture characteristics of sandstone

**Figure 11:**
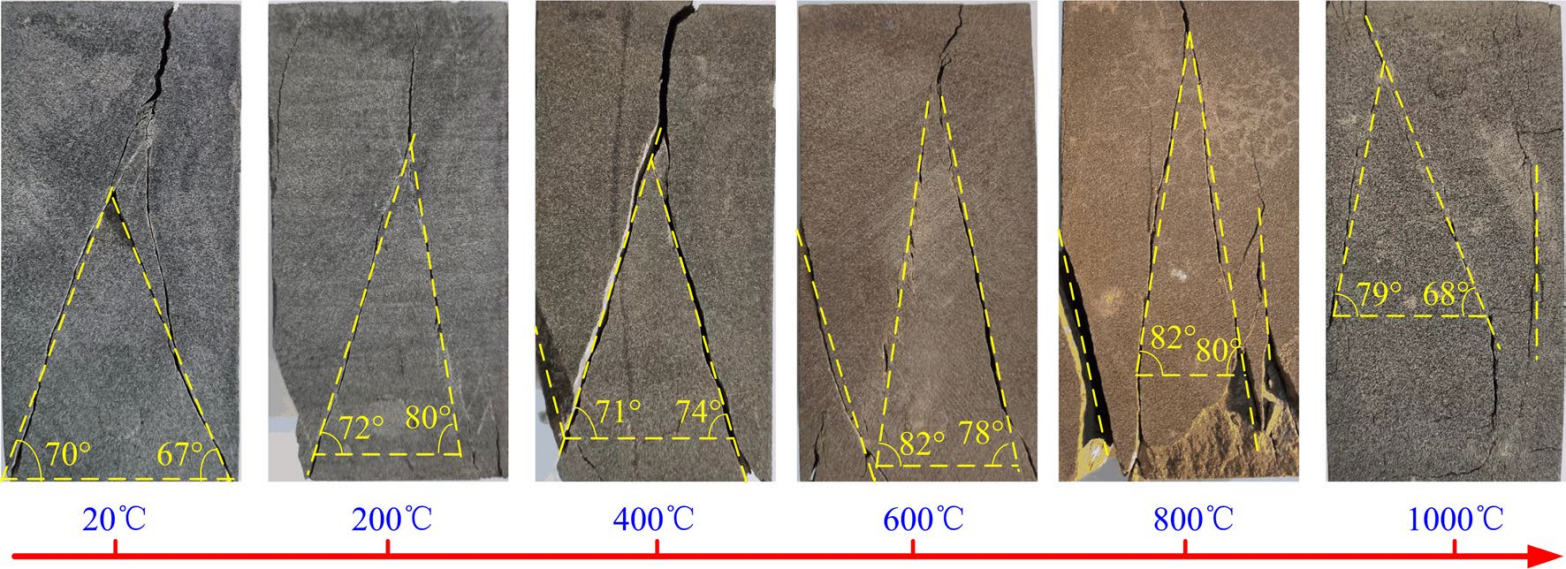
Damage pattern of specimens at different temperatures.

The failure modes of sandstone specimens under true triaxial conditions at different temperatures are shown in Fig. 11.

The failure modes of the specimens at different temperatures are similar to those of other true triaxial loading^49,53,60,61^, showing an inverted “Y” or an inverted “N”(see Fig. 11). With the increase in the maximum principal stress, the shear stress and the tensile strain parallel to the minimum principal stress on the oblique section of the rock gradually increase. The failure of rock is the relative displacement between rock mineral particles under the action of shear stress or tensile strain on the oblique section. The failure mode of the rock is tensile failure or shear failure, and it depends on the shear stress or tensile strain on the section reaching its limit value.

When the temperature is below 400 °C, the failure mode of the specimen is shear failure, which is caused by the shear stress on the failure surface reaching the limit value. When the temperature exceeds 600 °C, some specimens show shear-tension failure, which is caused by the tensile strain along the direction of the minimum principal stress, reaching its limit value under the action of maximum principal stress. The failure surface is rough and parallel to the minimum principal stress action surface. When failure occurs, it is similar to rock burst and flake peeling takes place, which is a characteristic of tensile failure.

**Figure 12:**
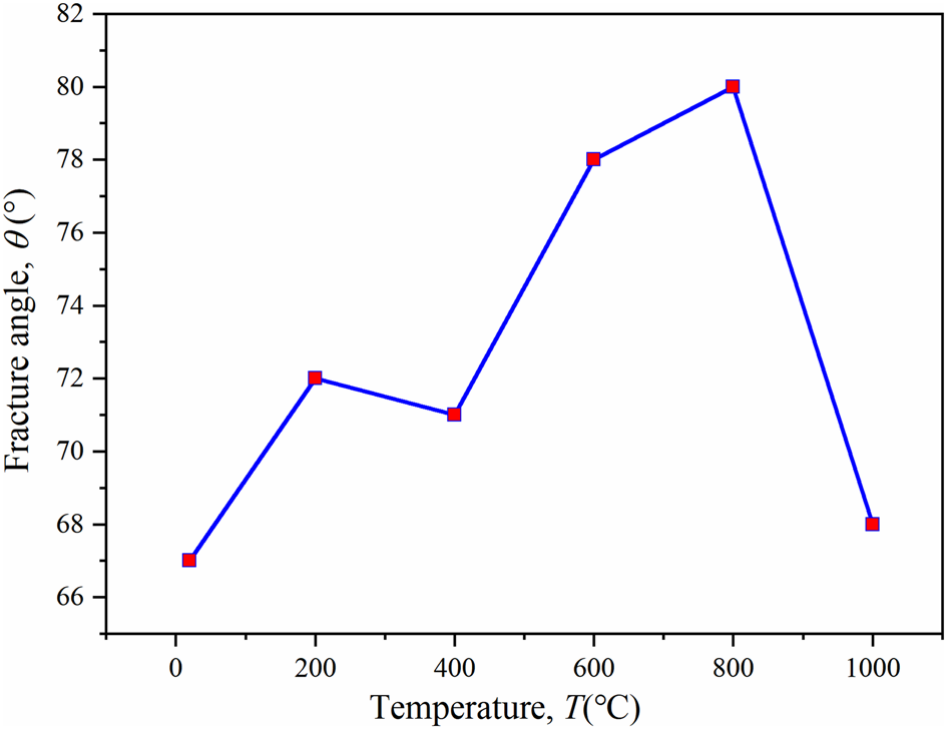
Minimum angle of fracture of specimens at different temperatures.

Generally, there are two failure cracks in true triaxial sample, and the minimum fracture angle is taken for analysis. The minimum rupture angle of the specimen at different temperatures is shown in Fig. 12. With the increase in temperature, the fracture angle of samples increases first and then decreases. When the temperature is 800 °C, the maximum rupture angle is 80 °C, and the rupture angle increases by 13 °C compared with normal temperature.

## Discussion

### Relationship between peak stress and temperature

**Figure 13:**
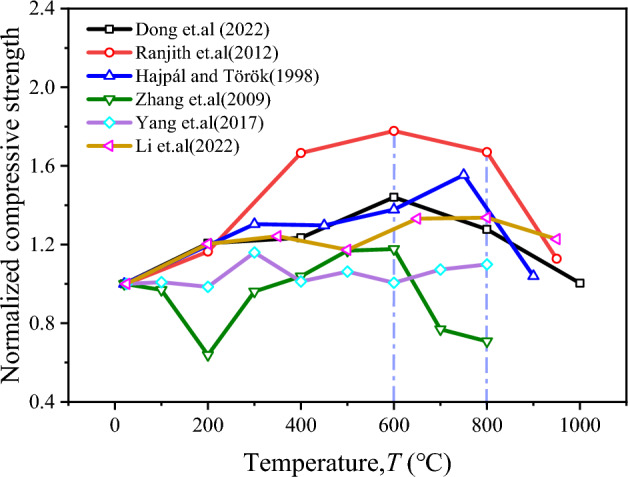
Peak strength of high-temperature sandstone as a function of temperature.

**Figure 14:**
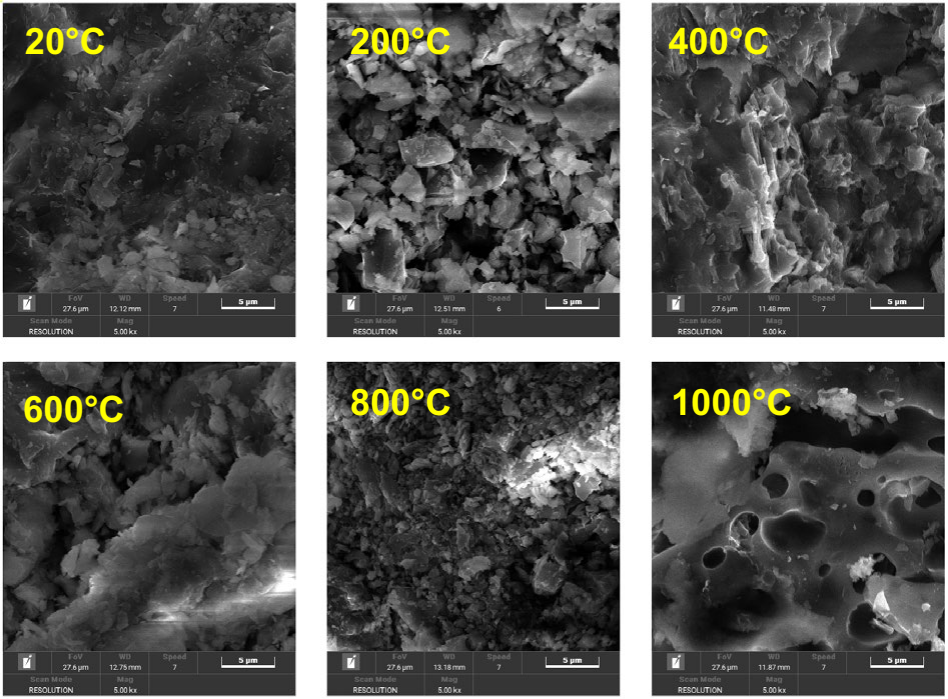
SEM image of high temperature sandstone.

The mechanical properties of high-temperature sandstones have been extensively studied by various researchers (Fig. 13). Hajpál and Török^62^ investigated the Cottaer sandstone in Germany and found a threshold temperature of 750 °C for the uniaxial compression strength. Zhang et al.^32^ conducted real-time high-temperature uniaxial compression tests on Xuzhou sandstone and identified a threshold temperature of 600 °C. Dong et al.^15^ and Ranjith et al.^22^ also observed similar peak strengths corresponding to threshold temperatures in their studies. Yang et al.^28^ performed conventional triaxial tests on Rizhao sandstone and found a threshold temperature of 300 °C with a peak strength of 10 MPa under peritectic compression. Li et al.^42^ conducted true triaxial tests on Sichuan quartz-rich sandstone after high-temperature treatment and observed a bimodal change in peak strength with increasing temperature, with a threshold temperature of 800 °C. The temperature thresholds for the bimodal variation are 400 °C and 800 °C in the text, while the sandstone at 800 °C represents the point of maximum strength in the single-peak variation. The effect of high temperature on sandstone strength is attributed to thermal stress resulting from mineral grain expansion and increased frictional resistance caused by dehydration. We observed a slight degradation in the mechanical strength of sandstone when subjected to temperatures of up to 600 °C. The peak stress decreased by approximately 15% compared to 400 °C conditions. Additionally, an increase in crack density was observed above 600 °C, primarily due to the α-β transformation of quartz. Furthermore, the study highlighted the influence of microstructural features (Fig. 14), including mineralogy and grain size distribution, on the high-temperature mechanical characteristics of sandstone. We found that the rock's microstructure played a crucial role in determining its response to thermal loading.

These findings further contribute to the understanding of how high temperatures influence the strength characteristics of sandstone and highlight the importance of considering both temperature and stress state in assessing its mechanical behavior.

### Temperature-moisture variation in sandstone

Based on the analysis of the above experimental results, the main mechanism of the change of sandstone mechanical properties after high temperature treatment includes water loss and structural damage caused by thermal reaction. The mass loss of sandstone is mainly manifested through the evaporation of water, and it is commonly characterized by the term “loss-on-ignition” (LOI). In this study, the LOI is defined as the ratio of the mass loss due to high temperature evaporation to the mass of the sample before heating, expressed as a percentage by Eq. (2).

2 $$LOI=\left[\left({m}_{s1}-{m}_{s2}\right)/{m}_{s1}\right]\times 100\%$$

where ${m}_{s1}$ is the initial mass of the sample before high-temperature testing, and ${m}_{s2}$ is the mass of the sample after heating and volatilization.

**Figure 15:**
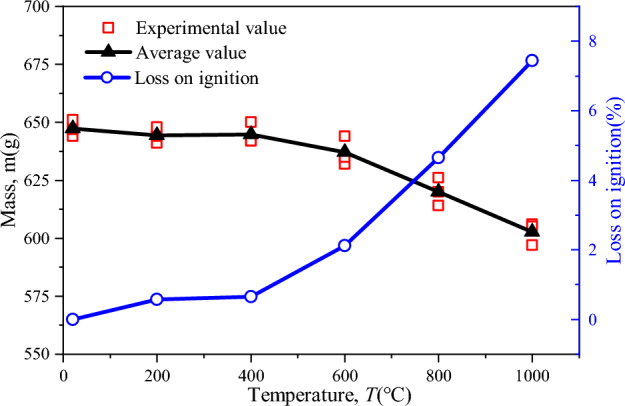
Relationship between mass loss of sandstone and temperature.

The relationship between sandstone mass loss and temperature is shown in Fig. 15. When the sandstone sample is heated to 200 °C, the quality of sandstone decreases slightly under the influence of thermal expansion and free water evaporation. Based on the quality of rocks after high-temperature treatment, the LOI of sandstone is 0.58%, and the overall rock mechanical parameters are improved due to the compaction of pores. At 200–400 °C, the LOI of sandstone is only 0.65%, which is very close to the results at 200 °C. When the sandstone sample is heated to 400–600 °C, some minerals in the sandstone are ablated, the bound water in the sandstone is lost, and the defects increase. In addition, organic matter such as carbon and sulfur will undergo thermal decomposition and other thermochemical reactions, which will further reduce the rock quality. The LOI of sandstone is 2.11%, which is about 3.7 times of the LOI at 200 °C. At the same time, as the temperature approaches 600 °C, the quartz transforms from the *α* phase to *β* phase^59^, during which the volume expands slightly and impurities in the quartz crystal are purified. This transformation causes great changes in the physical and mechanical properties of sandstone, which shows that the peak compressive strength of sandstone decreases by about 15% compared with 400 °C. At the stage of 600–800 °C, many minerals in sandstone begin to melt and phase changes occur, the LOI further increases to 4.65%, some minerals are ablated, the rock quality decreases continuously. When the sandstone sample is heated to 800–1000 °C, some minerals in the sample melt, resulting in many microscopic defects in the rock, among which the thermal reaction of clay minerals is enhanced, minerals are rearranged, the edges and corners of mineral particles disappear, the morphology of pores and cracks changes obviously, and even macroscopic cracks occur. The true triaxial compressive strength of sandstone decreases rapidly, and the peak strength is close to the true triaxial strength at NT.

## Conclusions and suggestions

In this study, a series of true triaxial tests (TTT) were carried out on coal-measure sandstone with a self-developed true triaxial test system combined with acoustic emission (AE) monitoring, and the mass loss, deformation characteristics and failure modes of sandstone before and after heat treatment were systematically studied. The main results of this study can be summarized as follows:

1. The true triaxial mechanical properties of sandstone after high temperature treatment are closely related to temperature, and the peak strength, maximum principal strain, minimum fracture angle and elastic modulus all show bimodal changes, not single peak changes shown by uniaxial compression and triaxial compression. The following rules are observed: the first peak strength appears from RT to 400 °C, and the second peak appears from 600 to 1000 °C. When the temperature rises to 800 °C, the sandstone reaches the peak strength of the high temperature test. At the same time, the maximum principal strain, minimum fracture Angle and volume strain also reach the peak, and 400 °C is the transition temperature of the elastic modulus.
2. The true triaxial high temperature sandstone test results are in good agreement with the existing true triaxial theory and experimental results. The failure modes of samples at different temperatures are inverted “Y” or inverted “N”. Shear failure occurs when the temperature is below 400 °C, and shear-tension failure occurs when the temperature is over 600 °C. There are four periods of AE signaling, namely the quiet period, growth period, burst period and decline period, and the number of AE events corresponds to the deviator stress interval.
3. By changing the heat-treatment temperature of the rock, it can be found that the strength and physical properties of rock change substantially and irreversibly after heating. The average peak strength of the sandstone reaches its maximum value at 800 °C, which is an increase of 84.2% compared to room temperature. Compared to 400 °C, there is an increase of 9.8%. However, at a temperature of 1000 °C, there is a sharp decrease in peak strength, with a decrease of 49.2%.

## Data Availability

The data used to support the findings and results of this study are available from the corresponding author upon request.
